# P-585. Comparison of Air Sampling in the Emergency Department Waiting Room and Clinical Testing for COVID-19, Influenza, and RSV

**DOI:** 10.1093/ofid/ofaf695.799

**Published:** 2026-01-11

**Authors:** Meggie Griffin, Helena Ikenberry, Amy Ellis, Ari Machtinger, David O’Connor, Shelby O’Connor, Michael Pulia

**Affiliations:** University of Wisconsin-Madison School of Medicine and Public Health, Madison, Wisconsin; University of Wisconsin Madison, MADISON, Wisconsin; University of Wisconsin-Madison School of Medicine and Public Health, Madison, Wisconsin; University of Wisconsin-Madison School of Medicine and Public Health, Madison, Wisconsin; University of Wisconsin-Madison, Madison, WI; University of Wisconsin-Madison School of Medicine and Public Health, Madison, Wisconsin; University of Wisconsin-Madison School of Medicine and Public Health, Madison, Wisconsin

## Abstract

**Background:**

Air sampling provides a novel approach for respiratory virus surveillance in the community including early detection of outbreaks and emergent pathogens. The emergency department (ED) is a unique environment where individuals with acute respiratory tract infections (ARTIs) receive treatment and potentially transmit viral illness to other patients waiting for care. Our objective was to evaluate the association between air sampling pathogen detection and the number of patients that tested positive for respiratory viruses in the ED.

**Table 1.**
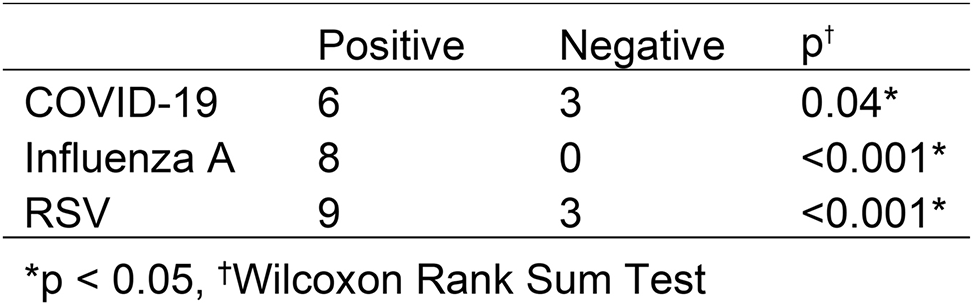
Median cases per time period by Cepheid GeneXpert air pathogen detection

**Figure 1.**
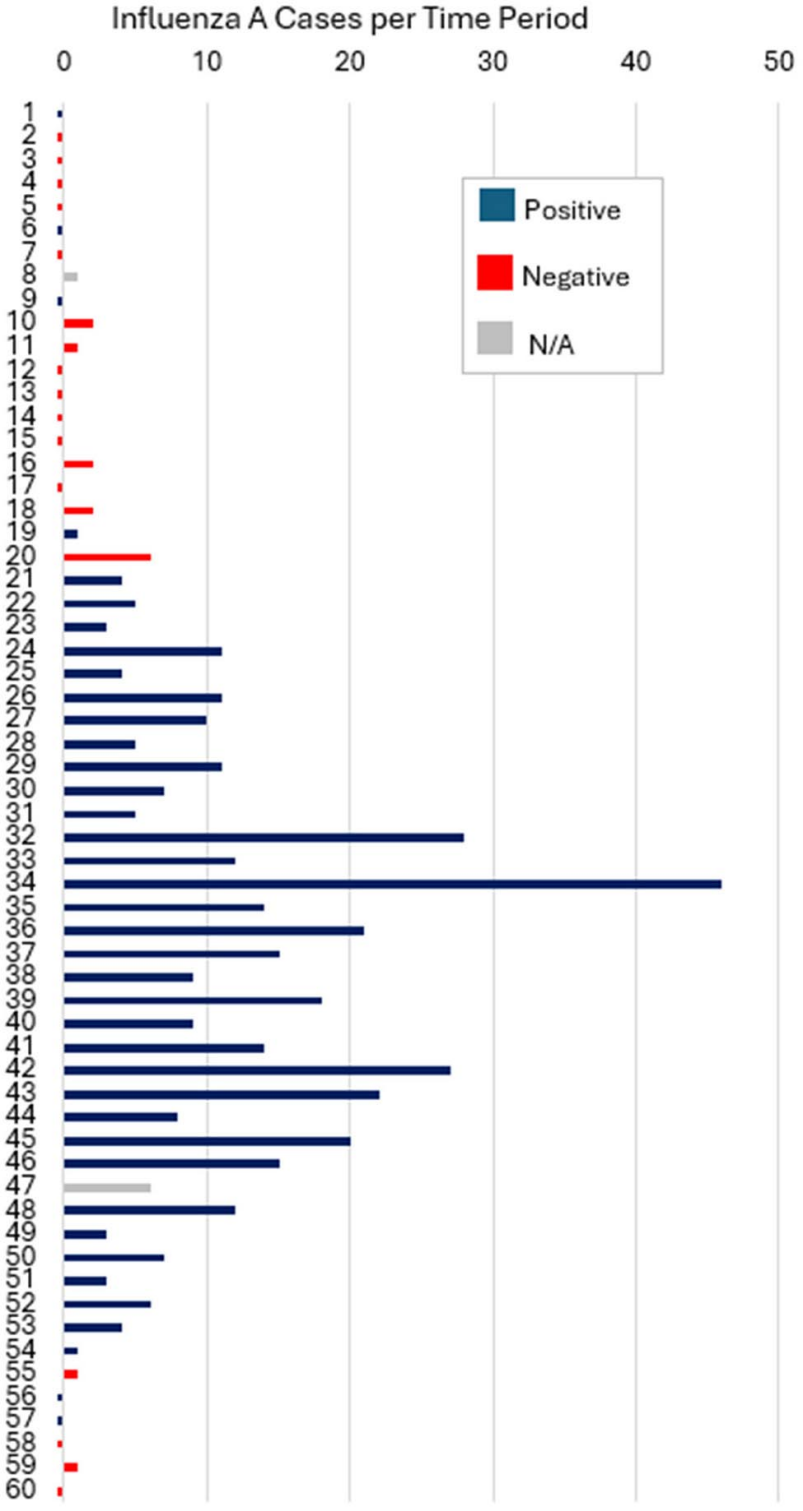
Number of influenza A cases for time periods (1-60) from October 24, 2024 – March 21, 2025 and air sampling pathogen detection by the Cepheid GeneXpert assay. All time periods were 2-3 days except for time periods (1, 22, 25, 32, 34) that were 4-5 days.

**Methods:**

We conducted surveillance of in-air pathogens in the ED waiting room at a Midwest quaternary care center from October 24, 2024 – March 21, 2025. The air sampler ran continuously, and cartridges were replaced approximately every 2-3 days for a total of 60 time periods across the study. Cepheid GeneXpert assays for COVID-19, influenza A, and respiratory syncytial virus (RSV) were run on each cartridge. For each time period the air sampler was running, we extracted respiratory viral testing results for ED patients with ARTIs from the electronic health record and found the number of positive cases for each pathogen. We grouped the time periods by air sampling result (positive, negative) and calculated the median number of cases per time period in each group. We used Wilcoxon rank sum tests to assess the difference in the distributions and median number of positive cases by group with a significance level of 0.05.

**Figure 2.**
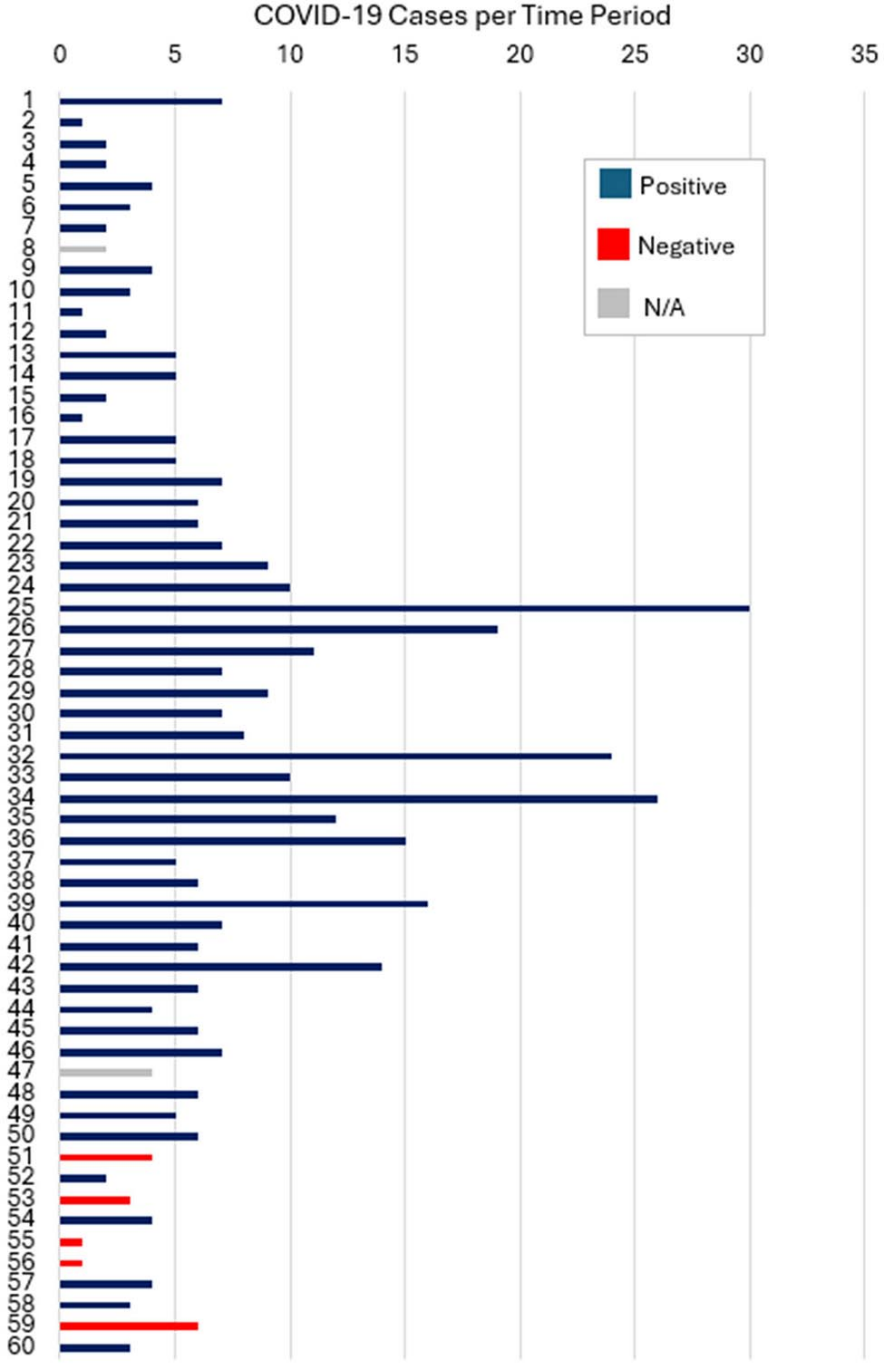
Number of COVID-19 cases for time periods (1-60) from October 24, 2024 – March 21, 2025 and air sampling pathogen detection by the Cepheid GeneXpert assay. All time periods were 2-3 days except for time periods (1, 22, 25, 32, 34) that were 4-5 days.

**Figure 3.**
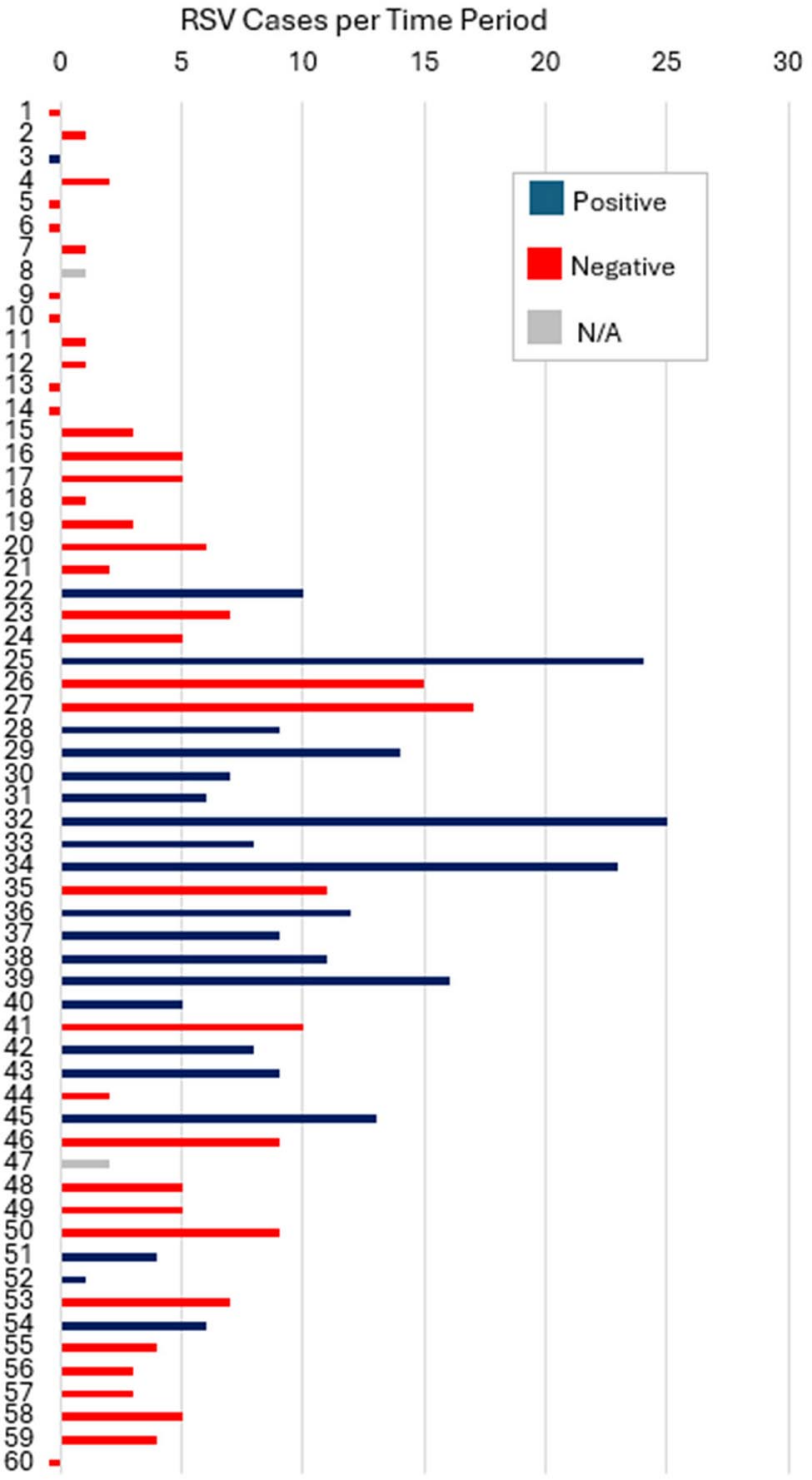
Number of respiratory syncytial virus (RSV) cases for time periods (1-60) from October 24, 2024 – March 21, 2025 and air sampling pathogen detection by the Cepheid GeneXpert assay. All time periods were 2-3 days except for time periods (1, 22, 25, 32, 34) that were 4-5 days.

**Results:**

We identified 408 COVID-19 cases, 413 influenza A cases, and 375 RSV cases. The number of cases by time period and the corresponding air sampling result are displayed for each pathogen in Figures 1 – 3. The median number of positive cases by positive vs. negative air sample results for COVID-19 (6 vs. 3 cases), influenza A (8 vs. 0 cases), and RSV (9 vs. 3 cases) were all significantly different (Table 1).

**Conclusion:**

Respiratory virus detection in ED waiting room air samples was associated with higher median positive tests for respiratory viruses in ED patients with ARTIs, demonstrating that air sampling surveillance correlated well with clinical caseload. Future work will compare ED air sampling surveillance with other community sites.

**Disclosures:**

All Authors: No reported disclosures

